# Tensile experimental data from monotonic loading on connections to concrete filled steel tubes using Extended Hollo-bolt blind bolts

**DOI:** 10.1016/j.dib.2024.111088

**Published:** 2024-10-29

**Authors:** Manuela Cabrera, Walid Tizani, Jelena Ninic

**Affiliations:** a Department of Civil Engineering, University of Birmingham, Birmingham, UK; b Department of Civil Engineering, The University of Nottingham, Nottingham, UK

**Keywords:** Extended Hollo-Bolt Blind Bolt, Concrete-filled steel tube, Tensile tests

## Abstract

This dataset is related to the research paper entitled “Fusion of Experimental and Numerical Data for Development of Extended Hollo-bolt Component Based Model” published in the Engineering Structures journal (Cabrera et al., 2024). It provides the necessary information for the collection of tensile load-displacement data from Concrete-filled Steel Tubes (CFST) connected to a rigid T-Stub using Extended Hollo bolts (EHB). The dataset encompasses (i) the load-displacement response of 28 EHB connections subjected to monotonic tensile tests, which include displacement data at the bolt head, bolt end, and column face; (ii) 40 pictures per sample taken at fixed intervals during the tensile testing, along with 2 pictures after failure, one before and one after careful removal of the top plate of the steel tube which facilitate the understanding of concrete damage and failure mode. This information is intended to provide insights into the failure mechanism of the EHB connections when components can interact and contribute to deformability of the connection as well as provide full data for development and validation of predictive models such as numerical, analytical, and data driven models.

Specifications Table

**Table:** 

Subject	Engineering; Civil and Structural Engineering
Specific subject area	Modelling of the behaviour of blind bolted connections in concrete filled steel tubes
Data format	Filtered data
Type of data	.csv files (dataset with measured load and displacement values) .jpg (sample pictures during testing and after failure)
Data collection	Load-displacement data was acquired by recording the Instron Universal Testing machine load cell and placing two strain measurement techniques in the samples: LVDT/linear potentiometers (POT) and a Video Gauge (VG) camera. Targets were placed at the bolt loaded end, unloaded end, and column face in order to record global displacement, bolt elongation and column face deformation respectively. In order to neutralize the effect of variations in the concrete strength on the day of testing, data was normalized by performing a nonlinear regression analysis of the six concrete samples. The recorded displacements were adjusted using the respective normalisation factor.
Data source location	Civil engineering laboratories, University of Nottingham, Nottingham, UK
Data accessibility	Repository name: Mendeley Data Data identification number: 10.17632/z9d3hw9szy.1 Direct URL to data: https://data.mendeley.com/datasets/z9d3hw9szy/1
Related research article	M. Cabrera, W. Tizani, J. Ninic, Fusion of Experimental and Numerical Data for Development of Extended Hollo-bolt Component Based Model, Engineering Structures (Accepted 03/09/2024)

## 1 Value of the Data

• This dataset comprises two components, namely (i) experimental results from tensile tests, and (ii) image recording of samples during the tensile experimental process. These components, particularly the images, can be valuable for detecting pixel-level changes using photogrammetry methods. Both can be useful for:
• Understanding the EHB connection when combined failure can occur, this is when all components (bolts, concrete, steel tube) interact and contribute to the deformability.
• Development, verification and validation of numerical and predictive mechanics models, such as finite element analysis and the component method.
• Development of data driven models for characterisation of EHB failure modes.
• Future research includes studying the group behaviour of the EHB connection with a wider range of parameter variation, full column-to-beam connections, and different loading conditions e.g. high strain rates, high temperature, cyclic, etc.

## 2 Background

Concrete-filled steel sections are popular in high-rise construction due to the steel tube providing concrete confinement and the concrete core restraining inward deformations of the tube. Blind fasteners in tubular steel columns offer sufficient tying and shear resistance for structural integrity checks but tend to have low moment-rotation stiffness due to the column face's flexibility [2]. Research on extended blind bolts in concrete-filled steel tubes is ongoing to develop moment-resisting connections [3].

This dataset was compiled to improve the understanding of EHB connections, particularly in terms of strength, stiffness, and ductility. While experimental studies often focus on the overall behaviour of connections, detailed information on the deformation of individual components is scarce due to the complexity of testing setups and spatial limitations. This dataset addresses this gap, providing comprehensive raw and visual data, including force-displacement curves, failure modes, critical for accurate modeling and design.

This dataset supports the original research presented in [1]. essential for validating analytical and numerical models, as well as for training machine learning models which rely on the availability of abundant and accurate datasets. This dataset enhances researchers’ understanding of EHB connections, contributing to more reliable design practices and the refinement of theoretical models.

## 3 Data Description

The dataset includes results from monotonic tensile tests were conducted on 14 pairs of Rigid T-Stubs connected to CFST using EHB blind up to failure using single-row (one pair of EHBs) or two-row (two pairs of EHBs) of bolts. The design variables considered are:

• Concrete strength C ($C_{des}$ corresponds to design value while $C_{test}$ is the concrete strength measured on the day of testing)
• Tube slenderness ratio μ (column width over thickness ratio μ=b/t)
• Bolt gauge distance g
• Bolt embedment depth $d_{emb}$
• Bolt grade BG
• Bolt diameter D
• Bolt pitch distance P

All tested steel hollow sections have a square section of 300 mm side. The benchmark samples correspond to specimens C40-1 & 2 were used as the specimen in the medium range of the investigated parameter for analysis of the effect of design parameter variation. The specimens numbering and design parameters are summarized in Table 1 where the unique test ID refers to the parameter of interest which differs from the benchmark.

**Table 1:** Test matrix and design parameter description.

Sample ID	$C_{des}$ (MPa)	$C_{test}$ (MPa)	μ	g (mm)	$d_{emb}$ (MPa)	BG	D (mm)	P (mm)
Effect of concrete strength C								
C20-1	20	20.5	30.0	140	86	8.8	16	-
C20-2	20	22.9	30.0	140	86	8.8	16	-
C40-1	40	41.6	30.0	140	86	8.8	16	-
C40-2	40	37.2	30.0	140	86	8.8	16	-
C80-1	80	77.8	30.0	140	86	8.8	16	-
C80-2	80	82.3	30.0	140	86	8.8	16	-

Effect of slenderness ratio μ								

µ47.6-1	40	49.8	47.6	140	86	8.8	16	-
µ47.6-2	40	36.4	47.6	140	86	8.8	16	-
µ30.0-1*	40	41.6	30.0	140	86	8.8	16	-
µ30.0-2*	40	37.2	30.0	140	86	8.8	16	-
µ18.8-1	40	38.0	18.8	140	86	8.8	16	-
µ18.8-2	40	36.2	18.8	140	86	8.8	16	-

Effect of gauge distance								

g80-1	40	45.2	30.0	80	86	8.8	16	-
g80-2	40	42.6	30.0	80	86	8.8	16	-
g140-1*	40	41.6	30.0	140	86	8.8	16	-
g140-2*	40	37.2	30.0	140	86	8.8	16	-
g180-1	40	36.9	30.0	180	86	8.8	16	-
g180-2	40	41.5	30.0	180	86	8.8	16	-

Effect of embedment depth $d_{\mathrm{emb}}$								

d_emb_66-1	20	36.2	30.0	140	66	8.8	16	-
d_emb_66-2	20	40.8	30.0	140	66	8.8	16	-
d_emb_86-1*	40	41.6	30.0	140	86	8.8	16	-
d_emb_86-2*	40	37.2	30.0	140	86	8.8	16	-
d_emb_106-1	80	38.7	30.0	140	106	8.8	16	-
d_emb_106-2	80	45.6	30.0	140	106	8.8	16	-

Effect of bolt grade BG								

BG8.8-1*	40	41.6	30.0	140	86	8.8	16	-
BG8.8-2*	40	37.2	30.0	140	86	8.8	16	-
BG10.9-1	40	38.1	30.0	140	86	10.9	16	-
BG10.9-2	40	43.1	30.0	140	86	10.9	16	-

Effect of bolt diameter D								

D16-1*	40	41.6	30.0	140	86	8.8	16	-
D16-2*	40	37.2	30.0	140	86	8.8	16	-
D20-1	40	36.5	30.0	140	86	8.8	20	-
D20-2	40	47.8	30.0	140	86	8.8	20	-

Effect of bolt pitch distance P								

P100-1	40	43.6	30.0	140	86	8.8	16	100
P100-2	40	41.7	30.0	140	86	8.8	16	100
P180-1	40	32.5	30.0	140	86	8.8	16	180
P180-2	40	32.2	30.0	140	86	8.8	16	180
P260-1	40	29.7	30.0	140	86	8.8	16	260
P260-2	40	29.9	30.0	140	86	8.8	16	260

⁎ Specimens tested as C40-1 & 2

The specific forms of data are:

*Tensile Data* – Normalised data are provided in .csv format under the “Data files” folder (see normalisation process in the following section). Each .csv file corresponds to a single tensile specimen and is named according to the corresponding sample. Files contain a header with metadata including the specimen's name, experiment date, and acquisition method. The remainder of each file contains the tabulated measured load displacement values, measured at bolt head (Pot1), bolt end (Pot2), and column face (LVDT1 and LVDT2).

*Images* – pictures of each specimen during testing and after failure are provided in .jpg format under the “Image files” folder, Each folder is labelled with the sample ID. After each test, a strip at the top face of the steel tube near the bolts was carefully cut and removed to examine the condition of the infill concrete, these images are used for the identification of the failure mode and are labelled as “SampleID-FAILURE”.

## 4 Experimental Design, Materials and Methods

### 4.1 Experimental Design

**Fig. 1:**
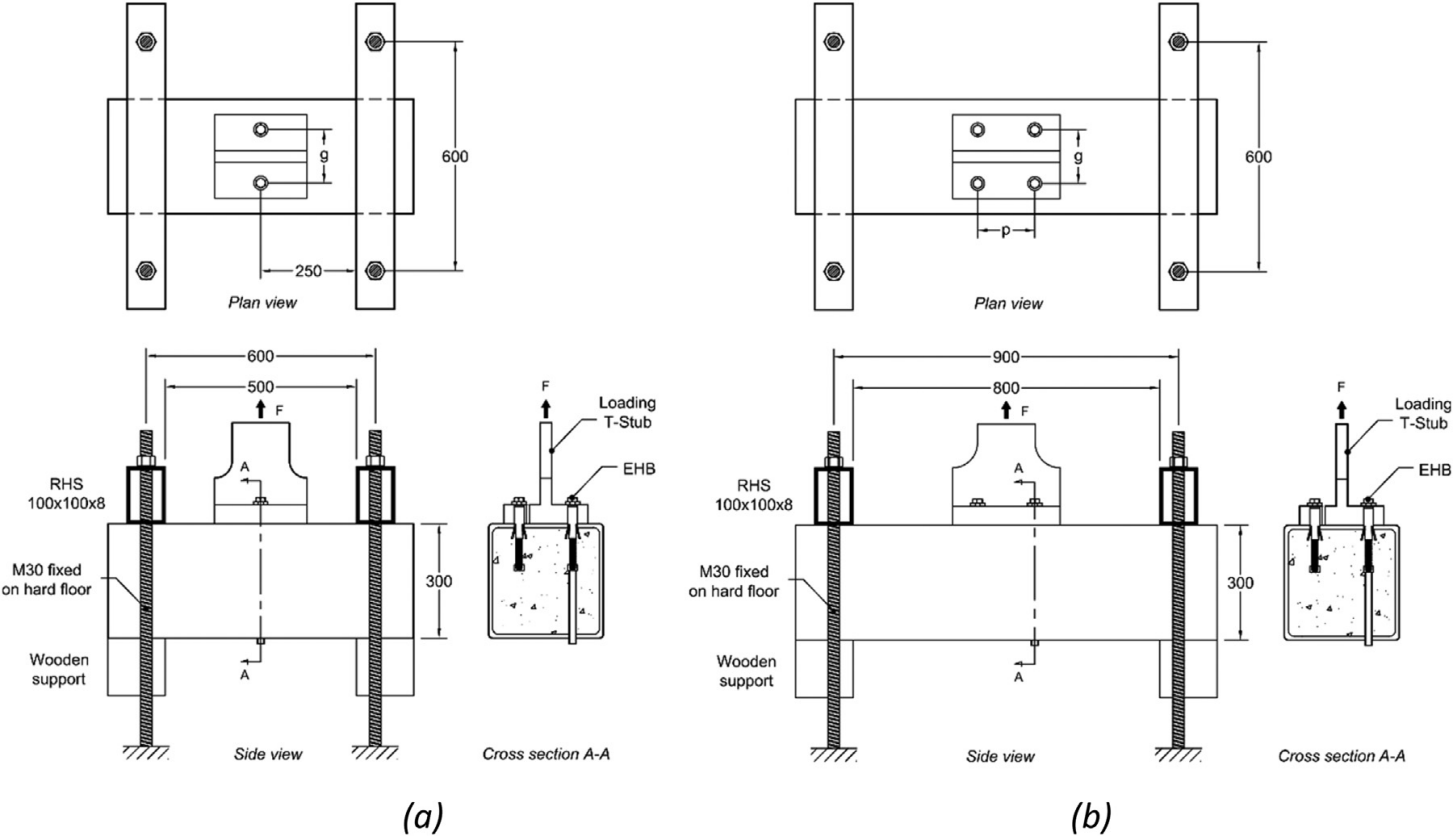
EHB tensile test setup for (a) one and (b) two rows of EHBs.

The test setup is illustrated in Fig. 1 (single EHB row in Fig. 1a, and double EHB row in Fig. 1b) involved a reusable T-Stub which allowed even distribution of the applied load to the connecting bolts. Reaction forces were provided by rectangular hollow section frames placed at both ends of the SHS at a distance bigger than two times$\;d_{emb}$ to allow the possible formation of a pull-out cone which is not influenced by the boundary conditions [4]. Wooden supports were used to provide access to the bottom of the sample to visualize the bolt extension.

The methodology adopted in the design of the test matrix is presented next. Three concrete grades C20, C40 and C80 were chosen to account for the low, normal, and high concrete strength ranges that are commonly used in construction.

**Fig. 2:**
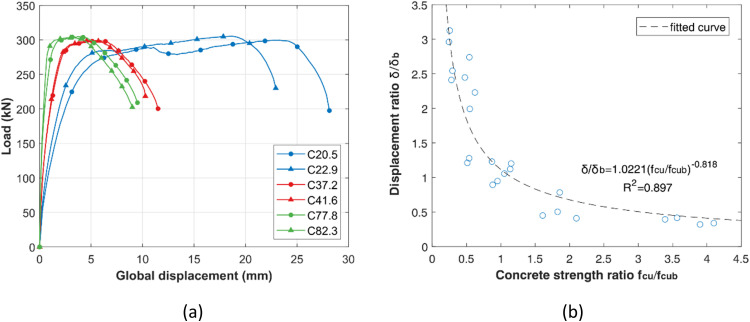
(a) Concrete strength effect on the global EHB connection behaviour (b) Concrete normalisation factor.

In order to neutralize the effects of concrete grade variation (see Fig. 2. (a) Concrete strength effect on the global EHB connection behaviour (b) Concrete normalisation factor. Fig. 2 (a)) in samples designed with constant C40 concrete strength, a normalization process was carried out by regression analysis: The 6 experimental samples were paired with each other generating the normalized concrete strength ($f_{cu}/f_{cub}$) and maximum displacement ($\delta /\;\delta_{b}$) ratios, where the *b* subscript denotes the base value. The ratios plotted in see Fig. 2. (a) Concrete strength effect on the global EHB connection behaviour (b) Concrete normalisation factor. Fig. 2 (b) approximate to a power function and best curve fitting was carried out producing Eq. (1). The recorded displacements were adjusted using the respective normalization factor $\delta_{n}$ calculated with this proposed equation for each of the following sections where the base was taken as C40.

(1) $$\delta_{n}=\frac{\delta \;}{\delta_{b}\;}=1.0221{\left(\frac{f_{\mathrm{cu}}}{f_{\mathrm{cub}}}\right)}^{-0.818}$$

Regarding the slenderness ratios, Tizani et al. [5] identified that EHB connections with column face slenderness ratios μ≤20 behave as rigid plates producing bolt shank failure. In order to investigate three failure modes of the connection, slenderness ratios μ=47.6, μ=30, and μ=18.8 were used in the present work to produce column face, combined, and bolt failure modes, respectively. This range also covers practical SHS sizes used in the building industry.

**Fig. 3:**
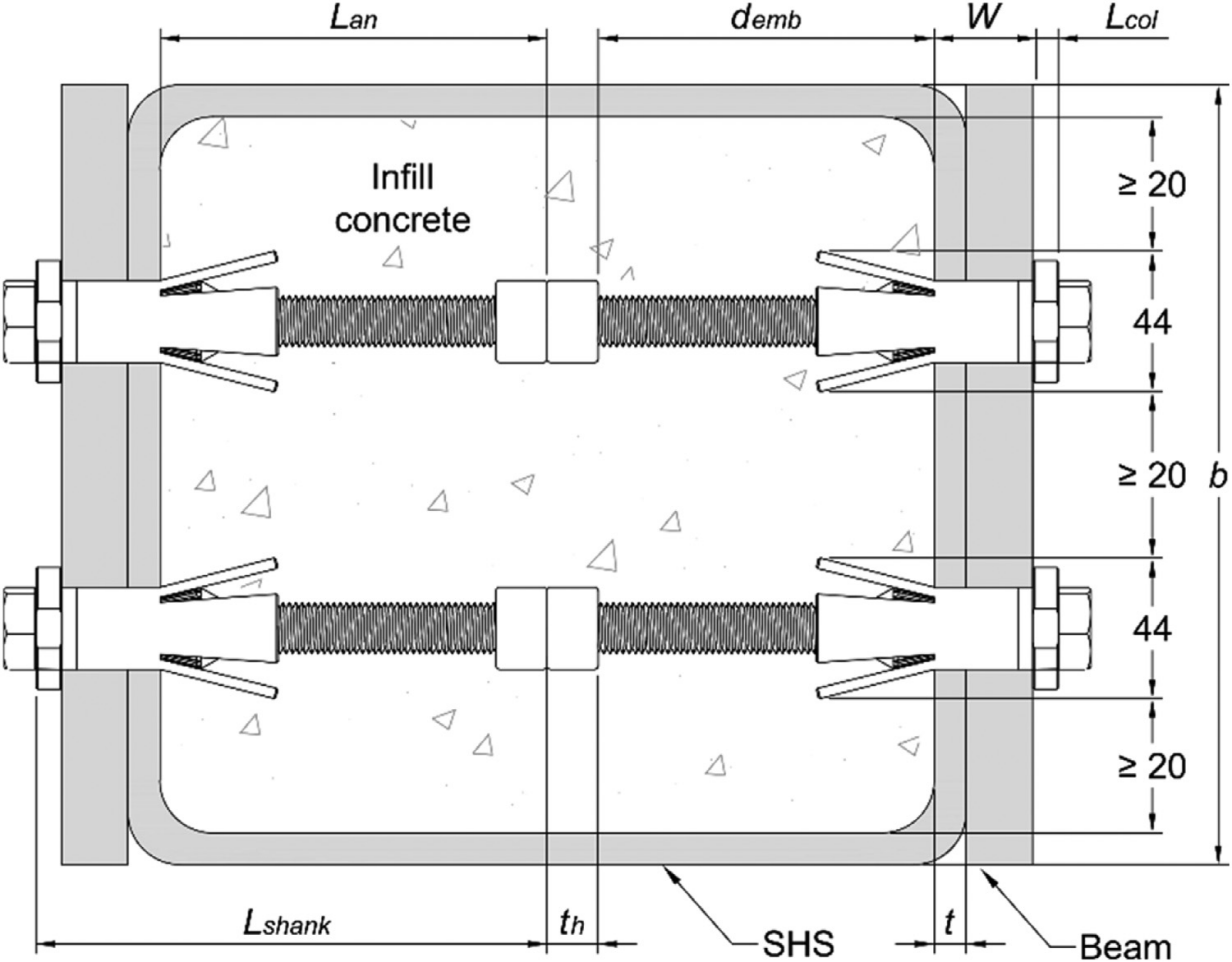
Spacing requirements for EHBs.

The minimum distance between EHBs depends on the opening size of the sleeves after tightening. The maximum opening width of the sleeves for a M20 bolt is approximately 44mm as illustrated in Fig. 3.

A minimum distance of 20 mm between sleeves was considered to account for the practical concrete placement requirements according to Eurocode 2 [6]. Therefore, the minimum bolt gauge ($g_{min}$) is calculated as $g_{min}=2x\frac{44}{2}+20=64mm$. Similarly, the minimum edge distance between the sleeves and the internal wall of the SHS should be bigger than 20 mm. Consequently, the maximum bolt gauge ($g_{max}$) for a square steel section with b=300mmis calculated using Eq. (2).

(2) $$g_{\max}=b-2t-\left(2x\frac{44}{2}+20\right)=216-2t$$

Where b is the steel section width and t is the steel section thickness.

The embedment depth ($d_{emb}$) is the distance between the inner SHS wall and the upper edge of the anchor nut (Fig. 3). It was calculated based on the minimum clamping width (W) of 50mm for a M16-3 sleeve [7]. The bolt anchored length and embedment depth are calculated using Eq. (3):

(3) $$\begin{array}{ccc} L_{an} & = & L_{shank}-L_{col}-W\\ L_{\mathrm{emb}} & = & L_{\mathrm{an}}-t_{h} \end{array}$$

Where: W: clamping width, $d_{emb}$: EHB embedment depth, $L_{shank}$: bolt shank length, $L_{an}$: EHB anchored length, $L_{col}$: collar thickness = 8 mm [7], $t_{b}$: Anchor nut thickness = Bolt diameter (d_b_).

The maximum anchored length can be calculated assuming that the column is connected to beams by EHBs from two opposite sides as from Eq. (4):

(4) $$g_{\max}=\;\frac{b-2t}{2}$$

## 5 Materials

The mechanical properties of concrete, steel hollow sections, and steel bolts, are summarized below.

Concrete cube sampling was carried out following the standard Testing concrete. Methods for mixing and sampling fresh concrete in the laboratory *BS 1881-125:2013* [8], and testing was performed according to *Testing hardened concrete - Compressive strength of test specimens BS EN 12390-3:2019* [9]. The concrete strength results on the day of testing are presented in Table 1.

The mechanical properties of the SHS were determined following the test procedure and coupon geometry in the standard *Metallic materials - Tensile testing - Part 1: Method of test at room temperature BS EN ISO 6892-1:2019-Annex D* [10]. The average material properties are reported in Table 2.

**Table 2:** Structural hollow sections material properties.

SHS dimensions	Yield stress $f_{y}\;$(MPa)	Ultimate stress $f_{u}\;$(MPa)	Young's modulus Es (GPa)
300×6.3	365.2	481.1	224.7
300×10.0	361.2	494.5	204.9
300×16.0	344.7	489.6	209.1

Machined bolt pieces were designed in accordance to the *standard Mechanical properties of fasteners made of carbon steel and alloy steel — Part 1: Bolts, screws and studs with specified property classes BS EN ISO 898-1:2013* [11]. The geometry and mechanical properties of the tested bolts are summarised in Table 3.

**Table 3:** Bolts geometry and mechanical properties.

Bolt type	M16-8.8	M16-10.9	M20-8.8
Nominal diameter (mm)	16.0	16.0	20.0
Property class	8.8	10.9	8.8
Sleeve diameter (mm)	26.0	26.0	33.0
Sleeve length (mm)	84.0	84.0	76.0
Collar thickness (mm)	7.9	7.9	10.0
Machined diameter (mm)	12.5	12.5	15.5
Machined stress area (mm2)	122.7	122.7	188.7
Yield stress fyb (MPa)	948.3	1191.2	847.2
Ultimate stress fub (MPa)	981.8	1266.5	966.5
fyb/Rp,02*	1.48	1.27	1.28
fub/Rm*	1.23	1.22	1.16

⁎ Where Rp,02 and Rm are stress at 0.2 % elongation and Tensile strength requirements according to BS EN ISO 898-1:2013 [11].

## 6 Methods

To ensure data reliability, two independent measurement techniques were used: linear potentiometers (POTs)/LVDTs for 1D displacements, and a Video gauge (VG) camera for 2D.

*POTs* - Two standard linear potentiometers were used to record displacements, one was located at bolt head or loaded end, and the other was placed at the bolt end or unloaded end. In order to record the displacement at the unloaded end, a threaded M16 rod was welded to one of the anchor nuts similar to research in [4]. This ensures the nuts, and the threaded rod remain in the correct position during concrete casting and vibration. The threaded rod extended to the outside of the SHS through a hole drilled at the back face. To eliminate any interaction between the concrete and rod, a 20 mm PVC conduit was used to cover the threaded rod. This tube was removed after concrete hardening. The unloaded end displacement represents the slip of the test bolt ($\delta_{slip}$), whereas the loaded end is the global displacement of the test bolt ($\delta_{global}$). The global displacement is thus comprised of slip plus elongation of the bolt shank $\delta_{b}$, resulting in $\delta_{global}=\delta_{slip}+\delta_{b}$.

*LVDTs* - Due to the limited space to monitor the displacement of the column face, two LVDTs were installed though holes pre-drilled in the T-stub. LVDT3 was located at 3 mm from the pre-drilled bolt hole, and LVDT4 at 50mm.

*VG camera* - The VG is a non-contact video-based system which tracks the displacement targets at different locations simultaneously. Five targets were identified to be monitored during the test: loaded and unloaded bolt ends, column face next to the bolt head, column edge, and sample movement. Contrary to the potentiometers, the whole sample displacement measurement is required in the VG system as the sample could move upwards during the application of the load while the camera is steady. This value was subtracted from other displacements to find the real column and bolt displacements. POTs and VG camera measurements were shown to be similar. POT data was therefore used for the analysis as it gives column face displacement values which could not be observed by the VG camera.

**Fig. 4:**
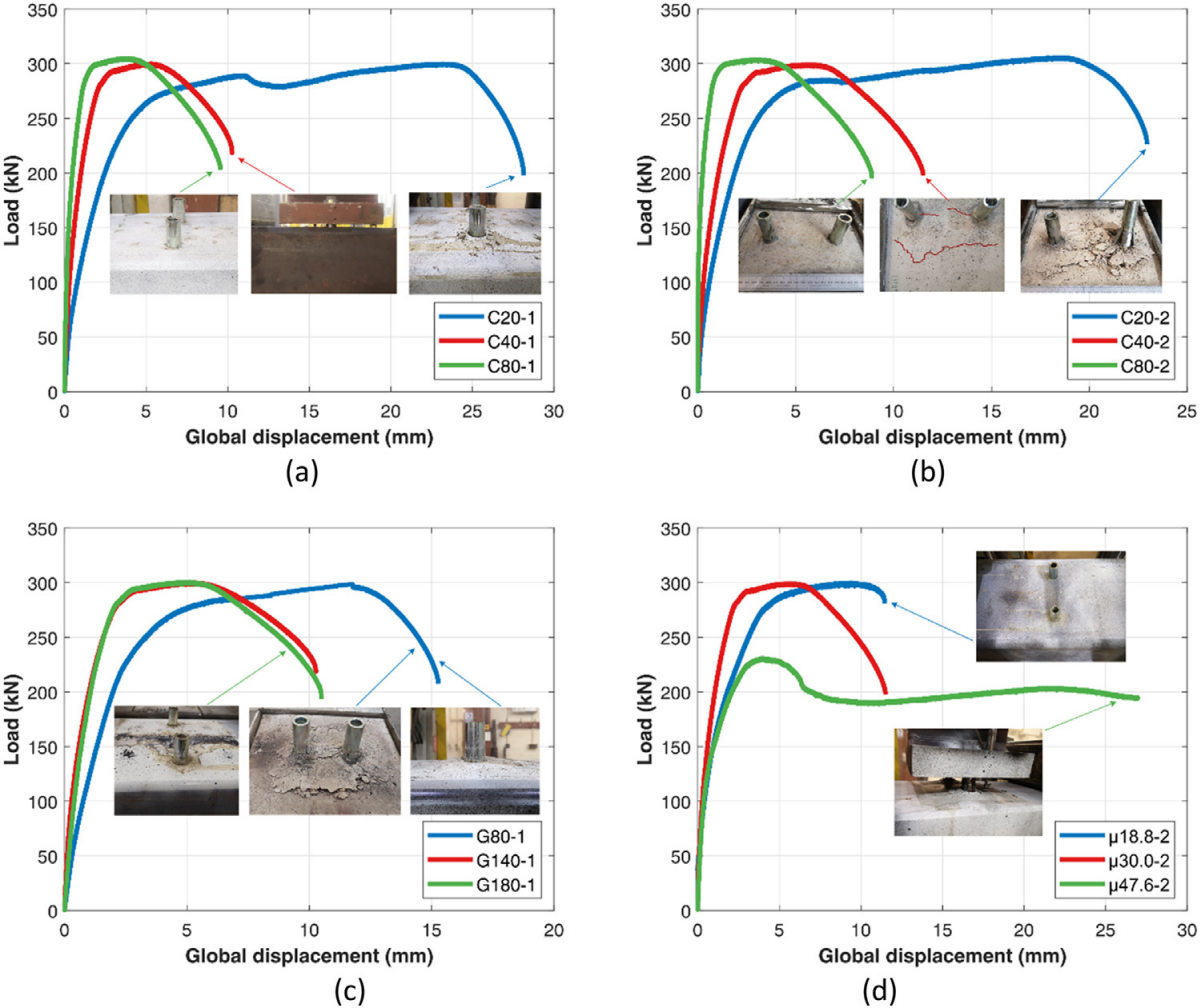

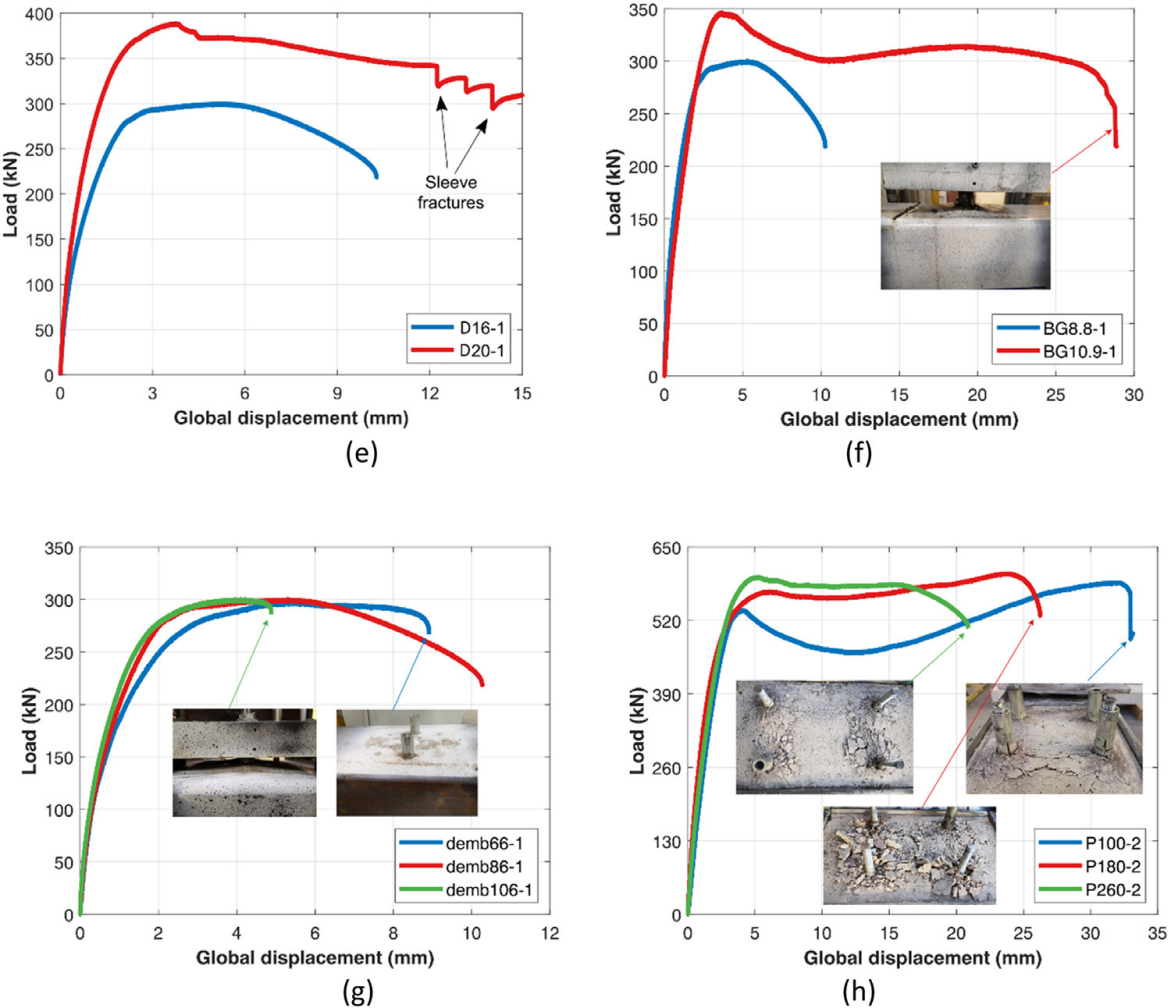
Load- global displacement curves with failure pictures for (a) (b) concrete, (c) gauge distance, (d) slenderness ratio (e) bolt diameter (f) bolt grade (g) embedment depth (h) pitch distance.

All tests were conducted under monotonic loading conditions at a rate of 0.015 mm/s until to failure, with the load- global displacement curves and corresponding failure images summarized in Fig. 4.

## Limitations

The following limitations were encountered while collecting the data:

• The data from column deformation was not recorded using the VG camera system as the visual of the area of interest is restricted by the T-Stub flange. This information was only recorded by POTs inserted through predrilled holes in the T-Stub.
• The photographs for sample D20-1 were not taken for the full duration of the test due to malfunction of the SLR camera.

## Ethics Statement

Data collection and processing were performed following the relevant institutional and national regulations and legislation and the ethical guidelines of Data in Brief. The authors declare that the current work does not involve human subjects, animal experiments, or any data collected from social media platforms.

## CRediT authorship contribution statement

**Manuela Cabrera:** Methodology, Investigation, Formal analysis, Writing – original draft. **Walid Tizani:** Conceptualization, Writing – review & editing, Supervision. **Jelena Ninic:** Writing – review & editing, Supervision.

## Data Availability

Mendeley DataDataset: Tensile experimental data from monotonic loading on connections to concrete filled steel tubes using Extended Hollo-bolt blind bolts (Original data).
